# Absorbing the gaze, scattering looks: Klimt’s distinctive style and its two-fold effect on the eye of the beholder

**DOI:** 10.16910/jemr.13.2.8

**Published:** 2020-10-06

**Authors:** Anna Miscenà, Jozsef Arato, Raphael Rosenberg

**Affiliations:** Department of Art History, University of Vienna, Austria; Vienna Cognitive Science Hub, University of Vienna, Austria; MECS, Leuphana University Lüneburg, Germany

**Keywords:** Eye movement, eye tracking, fixations, art perception

## Abstract

Among the most renowned painters of the early twentieth century, Gustav Klimt is often associated – by experts and laymen alike - with a distinctive style of representation: the visual juxtaposition of realistic features and flattened ornamental patterns. Art historical writing suggests that this juxtaposition allows a two-fold experience; the perception of both *the realm of art* and *the realm of life*. While Klimt adopted a variety of stylistic choices in his career, this one popularised his work and was hardly ever used by other artists. The following study was designed to observe whether Klimt’s distinctive style causes a specific behaviour of the viewer, at the level of eye-movements. Twenty-one portraits were shown to thirty viewers while their eye-movements were recorded. The pictures included artworks by Klimt in both his distinctive and non-distinctive styles, as well as other artists of the same historical period. The recorded data show that only Klimt’s distinctive paintings induce a specific eyemovement pattern with alternating longer (“absorbed”) and shorter (“scattered”) fixations. We therefore claim that there is a behavioural correspondence to what art historical interpretations have so far asserted: The perception of “Klimt’s style” can be described as two-fold also at a physiological level.

## Introduction

**Figure 1.**
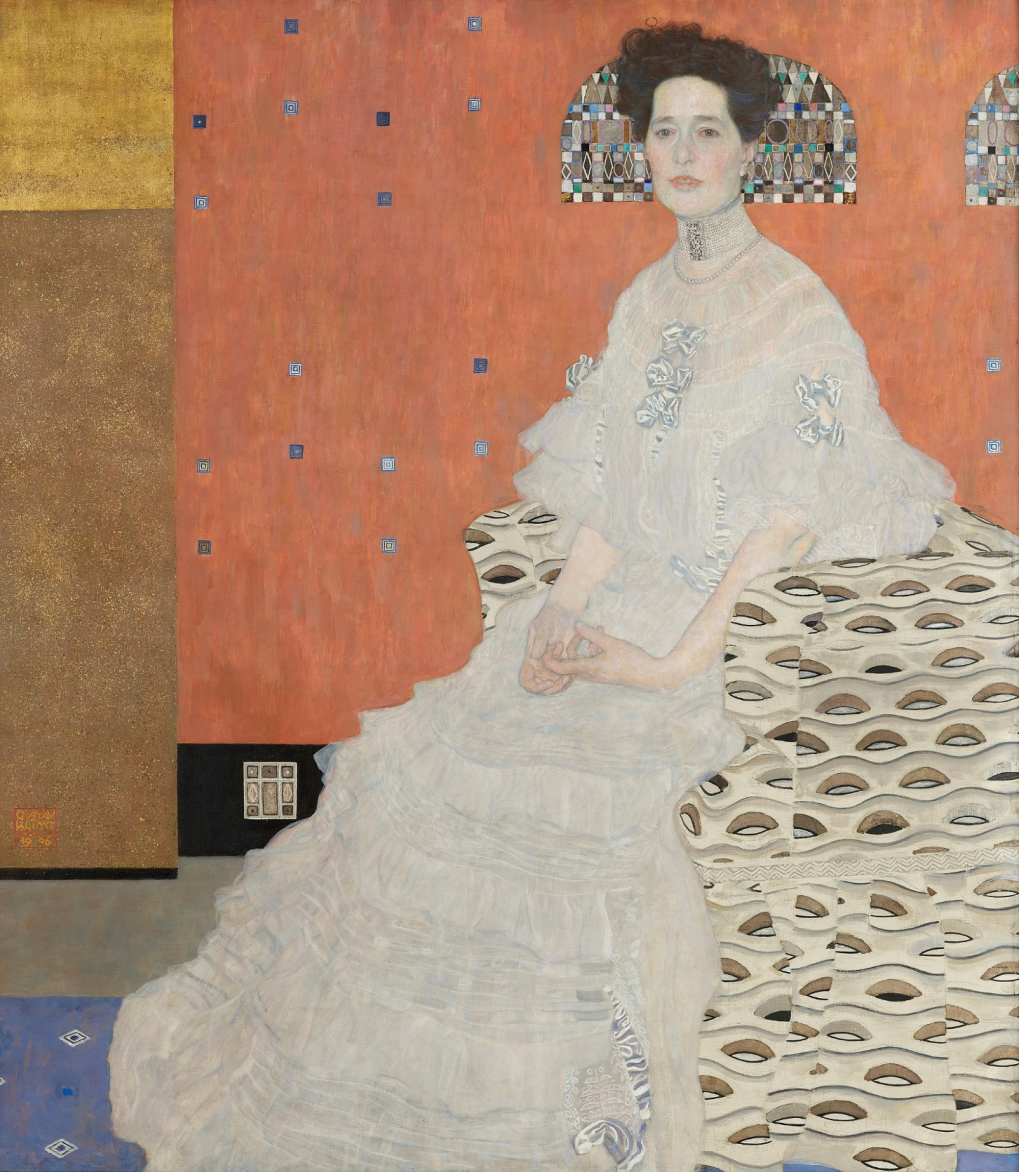
Gustav Klimt, Portrait of Fritza Riedler, 153 x 133 cm, 1906; Galerie Belvedere, Vienna

Not much is known about the life of Fritza Riedler, whom Gustav Klimt (1862-1918) painted in 1906 (Figure 1). Her portrait, today exhibited at the Belvedere Museum in Vienna, is perhaps just as enigmatic: The picture shows an elegantly dressed woman sitting on an armchair, smiling at the viewer. No detail is given about the space she is in. Orange brushstrokes, golden leaves, precious stones make up the paintings’ background; none of her, or her family’s belongings appear in the image. Fritza’s armchair is well visible in the foreground but it does not look like a piece of furniture; it resembles a bidimensional illusion. Instead of fabric covering the chair, eye-shaped gold and silver motifs seem to float on the surface of the painting. This ornament is flat, repeated irregularly, at times broken. It respects no rule of perspective. Its geometric shape suggests it has a symbolic nature; nonetheless, the woman’s face and plastic posture leave no doubts: We are looking at a real person.

Although we know relatively little about the women who sat for him, female portraiture played a key role in the career of Gustav Klimt. Portraits were usually commissioned by the family of the sitter, mainly members of the Viennese bourgeoisie; Klimt was highly remunerated for them. As they were also exhibited in public, these pictures could bring a great deal of prestige to Klimt’s female clients: Their names and faces would in fact be recognised by the distinguished audience attending art exhibitions. Photographs of the time prove that the sitters are always very recognisable; In 1902 Klimt completed his Portrait of Emilie Flöge, Viennese designer and socialite (1). The likenesses of Flöge made at the photo studio of Mme d’Ora show us just how convincing Emilie’s features are in Klimt’s (2). Her embroidered dress, however, was also turned into a geometric pattern much like Fritza’ furniture.

The decision of incorporating bidimensional elements was not dictated by the lack of artistic skills. Klimt had already realised several pictures which were overall realistic; it is the case of the *Portrait of a Lady in Black* or Marie Breunig (1894). The sitter is here shown in profile, posing with an elegant dress, her arm leaning on an upholstered chair; the whole image is realistic to the point of resembling a photograph. The furniture in this painting makes a fine comparison between a traditional, sober depiction and the way Fritza’s belongings were portrayed. In the light of this example, we can understand how the juxtaposition of pictorial tridimensional features and bidimensional ornaments in the second picture was a deliberate stylistic choice; and one which made Klimt earn a name for himself. In the first decade of 1900 Klimt produced some of his most renowned works, in which flat ornament is in strike contrast with realistic face and bodily features of the depicted figures; it is the case of *Judith* (1901) and the most notorious *The Kiss* (1907). In 1908, at the peak of his popularity, Klimt displayed Fritza along the portraits of Emilie Flöge and Adele-Bloch Bauer (1907) at the *Kunstschau* exhibition (3). In this occasion, art critic Joseph A. Lux claimed that women portrayed by Klimt could “rise above the ordinary” and become noble, unattainable, mysterious due to the unreal ornamentation surrounding them (1).

Klimt’s stylistic choices were considered unique at the time; today, they remain an exception in the artistic panorama of the twentieth century. Art-historically speaking, they can be located between two modes of representation. The first is traditional: Before 1900 the dresses and furniture stating social class of the sitter were depicted realistically, as in the case of Marie Breunig. The second one is modern; by the first decade of 1900, modern painters abstracted both the surrounding space and the sitter’s features to extreme flatness, often enough to turn them into visible brushstrokes. Klimt’s himself started adopting this mode of representation shortly after 1910. His most notorious and discussed pictures however remain the ones encompassing both bi- and tridimensional elements, between traditional and modern. Because this style was so short-lived and no other artist attempted to imitate it, the coexistence of realistic faces and flat ornament is something we still identify as being typical or distinctive of Gustav Klimt.

Art historians have discussed the effect of this coexistence in aesthetic terms. Many are the interpretations which followed that of Joseph Lux after 1908: The power of Klimt’s style continues to raise interest to this day. According to (4) “as the eye journeys across them, contemplating the juxtaposition of geometric shapes, and motifs inspired by nature (..) the inner life of the seemingly inert proclaims itself in its full glory”. The coexistence of bi- and tridimensional elements in Klimt’s paintings is said to evoke a peculiar aesthetic experience in the viewer; to “blur” the realm between art and life (4, 5, 1, 6). The specificities of such experience have also been described beyond purely aesthetic terms. Critical responses to Klimt’s exhibitions have claimed that his distinctive style holds the attention of the viewer for longer than other paintings, absorbing the gaze through the hypnotic repetition of patterns (7, 8). In order to explain some of the reasons we find these paintings attractive, neuroscientist Eric Kandel has asserted that “our brains assemble Gustav Klimt’s paintings piece by piece, symbol by symbol, tricking us into sensing the beauty of the whole” (9). Despite the large existing literature on the effects of Klimt’s distinctive style, spanning from academic writing, through exhibition reviews, to scientific interpretations, no research has been conducted on its physiological effects. We do not know whether the perception of these portraits really elicits a specific response in the viewer. Therefore, we designed a study in order to answer this question with an experimental investigation, conducted at the level of eye-movements.

Our investigation was designed to test the accuracy of two different claims drawn from art writing; the first, as reported in the above cited art historical literature, is that compared with other portraits Klimt’s distinctive style elicits a unique response in the viewer. The second is drawn from the contemporary criticism, again cited above: That this response implies “absorbing” the gaze consistently and visually “assembling” his paintings piece by piece; a behaviour which we theorised would be reflected by density of fixations (“absorbing”) and duration of fixations (“assembling”). The number of fixations is an indicator of visual attention directly influenced by top-down factors such as colour contrast and dynamism (10, 11). An “absorbed” gaze is therefore here intended as a series of fixations falling repeatedly in the same portion of an image. Fixation duration on the other hand has been shown to adjust to processing difficulty and to increase with increasing level of complexity in the perception of scenes (12, 13). Complexity of artistic images has been described as both a formal characteristic - the amount and variety of represented elements - and a semantic one - whether the iconography of a picture is usual or unusual ( 14). An “assembling” gaze is therefore intended as a series of long fixations, aimed at the identification of a subject matter or contextual information. In the case of Klimt, the art historical literature reported above suggests that the juxtaposition between realistic and flat elements enhances the ambiguous “unreal” character of the second in the image; thus we theorised that this choice leads us to explore ornament consistently and at length: First because it is visually striking, and secondly in order to understand the role it plays in the portrait.

**Figure 2.**
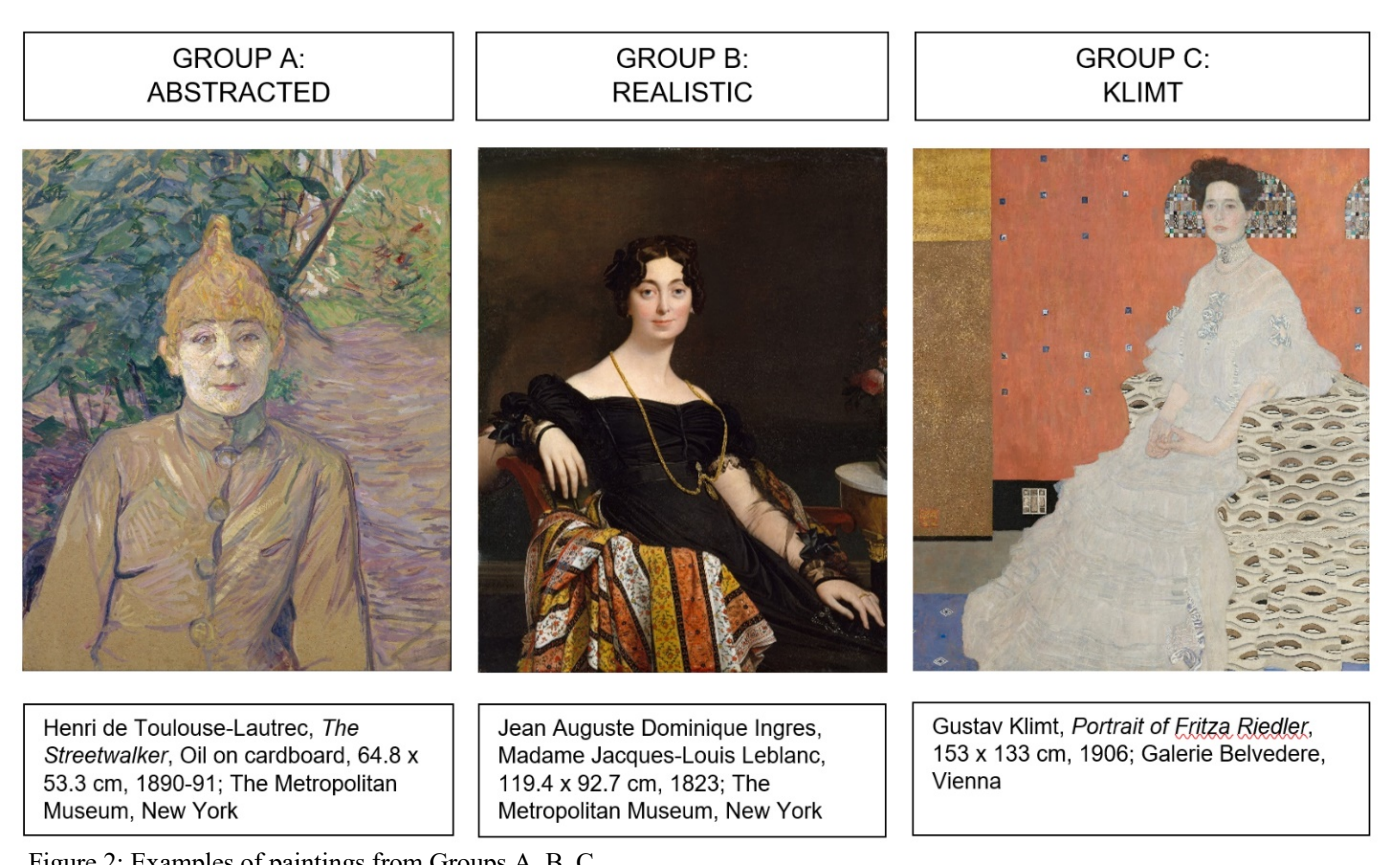
Examples of paintings from Groups A, B, C

To test these claims we selected seven of Klimt’s depictions of women and compared them with two other groups of female portraits including ornamental patterns, produced between 1823 and 1918. Reproductions of these paintings were shown to thirty participants (or “beholders”) in a laboratory setting in order to monitor their eye-movements. The stimuli were chosen and classified in three groups according to stylistic characteristics. Group A included seven modern portraits: These were paintings of artists such as Picasso and Van Gogh, known for their bidimensional treatment of the canvas. The ornament in these pictures consists of brushstrokes and juxtaposition of colour: Much like Klimt’s, in some cases it has no representational value; in others it is ambiguous enough to suggest a background or a setting. It is always, however, flat. While in this sense there are analogies with Klimt’s pictures, the painterly treatment of facial and bodily features by modern artists makes the contrast between bidimensional and tridimensional less evident – no clear discrepancy between the two is discussed in art historical terms. Group B included seven traditional portraits realised with a pictorial tridimensional style: Pictures in this group belong to the artistic trends of pre-1900. Both the sitters’ faces and their surroundings are here depicted realistically. The third group (C) contained seven portraits by Gustav Klimt, realised in his distinctive style (Figure 2). A more detailed description of the parameters of selection follows in the Stimuli section of this paper.

**Figure 3.**
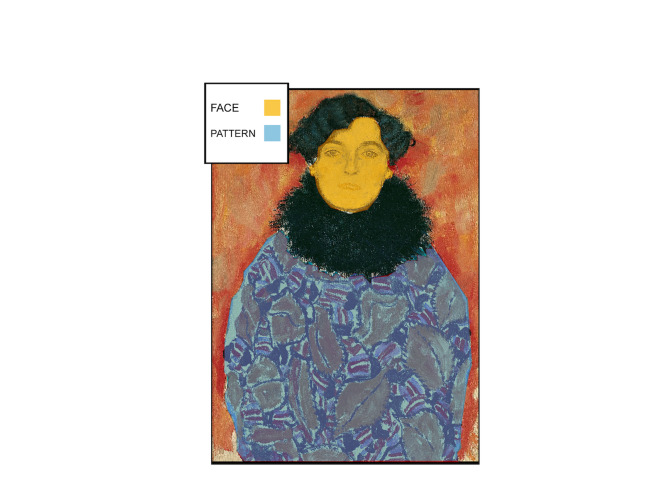
AOIs traced on Gustav Klimt, Johanna Staude (1918)

Since human beings are naturally drawn to look at faces (15) we assumed that looking at every portrait visual attention would have in any case been directed first and for the longest time to the face of the sitter. To find a monitorable difference between styles of representation we looked at the relationship between faces and ornamental patterns; we thus compared their effect across groups. For each painting we defined two Areas of Interest (AOIs) a priori: FACE, for facial features and PATTERN, for ornament (Figure 3). The two measurements used to monitor cognitive processing were the density of fixation and the average fixation duration within specific areas of interest. Given that we wanted to test whether Klimt’s style elicits a unique response, we imagined participants to have a comparable reaction to Group A and B and Group C or Klimt’s distinctive artworks to be looked at in a different way. We then formulated the following Hypotheses;

### Hypotheses

1) In Group A, B, and C the AOIs FACE would stimulate a higher density of fixation and a higher fixation duration than the AOIs PATTERN

2) AOIs PATTERN would be closely comparable for Group A and B both in terms of fixation density and average fixation duration. Given the fundamental stylistic differences between the two groups we anyway imagined a small discrepancy between them: AOIs PATTERN in Group A would stimulate a higher fixation density and lower average fixation duration than in Group B; in other words, participants would look consistently at abstracted patterns, colourful and dynamic, but for a shorter time, given their lack of complexity.

3) AOIs PATTERN in Group C would stimulate the highest fixation density and average fixation duration of all groups; people would look at Klimt’s patterns consistently and for a long time. If this last hypothesis was proven true, we would have been able to assert that Klimt’s style both “absorbs” our gaze on one side and leads us to “assemble” piece by piece his ornament, on the other.

## Methods

### Participants

Thirty participants were recruited in the Art History department of the University of Vienna through public advertisement in classes and seminars. (Range age: 18-30, average age 22,2 years). All participants identified as female, in concordance with their biological sex, and had normal or corrected-to-normal vision. All participants were bachelor students of Art History; they were naive to the purposes of the experiment but aware that they were going to be shown a series of artworks.

The main concern behind the criteria of selection was monitoring the behaviour of people who would visit a museum to see Klimt’s work. Bachelor Art History students were thus selected because of their explicit interest in art, but lack of specialisation at this point in their studies. Participants agreed to take part to the study for monetary compensation (5€ for ca. 30 min.).

### Stimuli

Twenty-one portraits of women (in high-quality digital reproduction) were used as stimuli. All the paintings presented traditional portrait orientation (height>width) and showed one figure, a female sitter, with visible and well identifiable facial features. We were mainly interested in monitoring the effect of the visual interaction between faces and ornament. For this reason, we chose portraits which included at least one visible ornamental motif or design. While all portraits could be described as similar in terms of composition and content, they differed in style and were thus classified according to stylistic rendering of the subject.

Art-historically speaking, style is not strictly measurable: it is a word which describes the choices consciously or unconsciously made by an artist in order to portray a certain subject. Whenever art historians define a style, they do so by comparing elements that are perceived as opposite between different artworks (For a classical example see (16); we define what is abstracted through comparison with something that is more representational, and vice versa. As such, the selection of each stimulus for this experiment relied on agreement between trained art historians. Our starting point were the parameters of Klimt’s *distinctive* style. As illustrated above, art historical literature covers these parameters extensively. Among Klimt’s artworks we thus selected only those where facial features were pictorially tridimensional and ornament completely flat, and labelled them as Group C. The two groups of comparative stimuli were then defined through similarity or contrast with this first selection; one more modern, the other more traditional than Klimt in their treatment of both features and ornament. Group A presented strong colour contrast and dynamic brushstrokes, as recurrent in modern art; Group B showed a realistic rendering, as typical of more traditional (pre-1900) art.

The formal parameters defining Groups A and B are not as clear-cut as those defining Group C. To anyone who is not used to this art historical exercise, the attribution of different paintings to two extra categories - traditional or modern - might feel unnecessary; we could have simply chosen to compare Klimt’s paintings to other female portraiture produced at any time in history. Nonetheless, it is precisely in respect to these categories of earlier or later forms of representation that Klimt’s distinctive style is intended as *unique* in the history of art. What our selection allows us to do is to test whether these claims of uniqueness remain valid in comparison with such loosely defined groups of material.

To reinforce the idea that the distinction between groups is based on formal aspects of style and not on the name of the artist alone, one painting by Klimt in non-distinctive style was included in each Group: *Lady in White* (1917), which shows no pictorial tridimensional features and very loose brushstrokes in Group A and the above mentioned *Lady in Back* in Group B. Stimuli were downloaded in high quality reproductions and high resolution from art historical databases (ARTStor library https://www.artstor.org) and from Museum collections online. A full list of the stimuli can be found in the Appendix section of this paper.

### Materials

The study comprised two subsequent phases: an eye-tracking experiment, followed by a short questionnaire. It was designed to last approximately thirty minutes. We presented the digital reproductions on a 2160 x 3840 BENQ LCD monitor, perpendicular to the viewer’s sight, using a maximum of 2880 pixel height and corresponding width. The eye movements of each participant over the stimuli were recorded using the EyeLink 1000 Plus remote eye-tracker at a 1000 Hz monocular frequency. The distance between the eyes of participant and monitor was set to 180 cm; the camera unit of the eye tracker were at approximately 50,5 cm distance from the participant’s eyes. The presentation sequence and randomisation of Stimuli was controlled by Experiment Builder, a software provided by SR Research for experiment design on the EyeLink 1000 Plus. The tracking session was initialized by a 13-point calibration and validation procedure to ensure a spatial resolution error of less than 0.9° of visual angle. The questionnaire’s main purpose was verifying the extent to which participants were familiar with Klimt’s artworks and aware of their defining stylistic features an assumption based on art historical literature, but also verifiable empirically. After being asked if they could identify any artwork and/or any artist in the sequence they had just been shown (“Among the paintings which have just been shown to you, was there any one you had already seen? If Yes please circle them” and “During the study, could you recognize any artist?”) people were asked if they could name one or more characteristic or element which they would identify as typical of the artist they recognized.

### Procedure

Participants were invited upon appointment to the CReA Lab at the University of Vienna, where they were informed that the study involved looking at a series of artworks on a computer screen and that the study’s main concern was investigating their appreciation of images. They were required to participate actively in the investigation. Participants were then asked for written consent in accordance with the Declaration of Helsinki and to anonymous treatment of personal data in accordance with regulations of the University of Vienna. We performed an Ishihara test in order to exclude dyschromatopsia and a sight test to ensure that each participant had normal or corrected-to-normal vision. We also assessed a Porta test to determine sighting dominance. Participants were then led to the Eye-tracker; they were instructed to sit in front of the screen, in straight position, and to avoid fast head movements. During the eye-tracking experiment, the twenty-one stimuli were shown for thirty seconds each, in randomised order. Participants were asked to view the stimuli as though they were in a museum. Each stimulus was followed by the question “How much did you like this painting?” to which participants had to answer on a Likert scale from 1 to 7. This question was not relevant for data analysis purposes but served to encourage participants to adopt an aesthetic attitude while looking at the artworks. Participants were not told their eyes were being tracked, so that the experiment could resemble a general survey concerning people’s appreciation of art.

Upon completion of the tracked session, participants filled in a printed questionnaire. Once they handed it over, they would be informed about the purpose of the study and remunerated for their participation.

### Data Analysis

The detection of fixations and saccades was performed with the Data Viewer software (SR Research). The events were then imported to Eyetrace ( 17) for the visualisation of fixations in Areas of Interest (AOI) and further analysis. We pre-defined AOIs semantically, identifying the portions of the picture which corresponded to the face of the sitter and to the ornamental pattern; when the latter was repeated in different portions of the image, each portion was identified as belonging to one single AOI. We manually traced AOIs during the analysis phase with Eyetrace’s AOI instrument. Average fixation durations were computed by EyeTrace for the AOI FACE and the AOI PATTERN for all participants across twenty-one paintings. The same process was followed to obtain the share of fixation in AOI (percentage). Fixation density was then calculated using Python dividing the share of fixation in AOI by the proportion of each AOI to the whole painting (in pixels). We run Paired t-test and repeated measures analysis of variance.

## Results

The questionnaires revealed that 27 out of 30 participants were able to recognise the work of Gustav Klimt and twenty-nine had previously seen his paintings. Of these, twelve named “Ornament, ornamental” as Klimt’s typical or defining characteristic; five mentioned “Patterns”, four “Geometrical motifs” and three “Decoration”. Among these twenty-four people, five mentioned the realistically rendered faces, and only one the contrast between the face and the ornamental designs.

Figures 4.1 and 4.2 show the fixation duration (ms) and density of fixations monitored across groups (A-B-C) and AOI type (Face vs Pattern). We performed a two-way repeated measures ANOVA. For fixation duration, there was a highly significant effect of AOI type (F(1,29)=54.23, *p*<.001) and painting group (F(2,58)=15.16, *p*<.001). There was also a highly significant interaction, suggesting that the effect of AOI type depended on the painting group (F(2,58)=14.61, *p*<.001). The same went for density of fixation; there was a highly significant effect of AOI type (F(1,29)=277.83, *p*<.001), of painting group (F(2,58)=61.8, *p*<.001) and a highly significant interaction (F(2,58)=37.58, *p*<.001).

**Figure 4.**
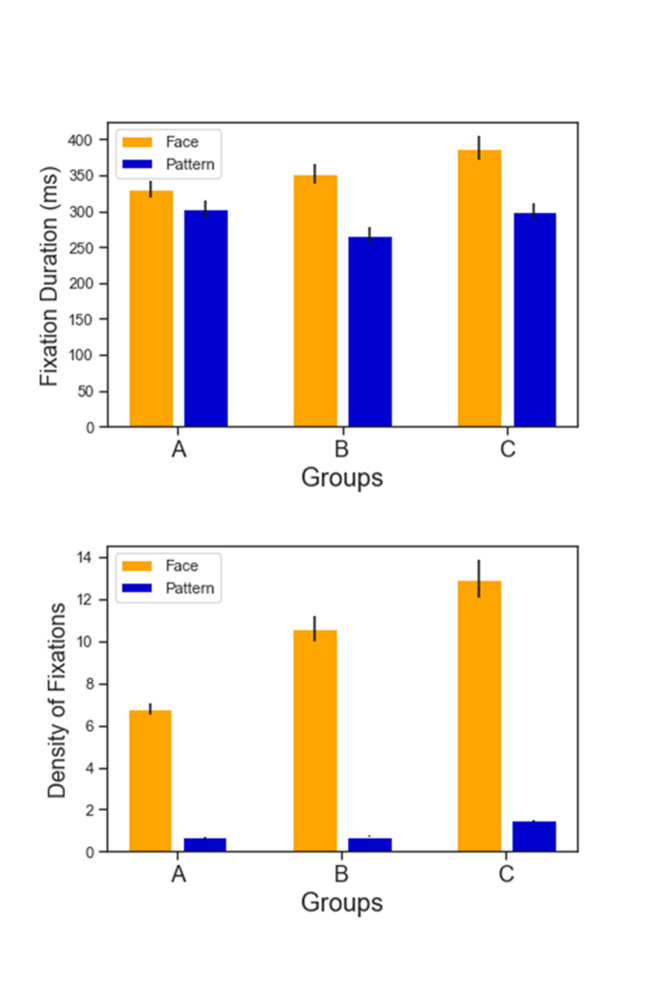
Top (4.1): Fixation duration (ms) across groups for AOI Face (Yellow) and AOI Pattern (Blue). Bottom (4.2): Density of fixations (ms) across groups for AOI Face (Yellow) and AOI Pattern (Blue)

Figures 5.1 and 5.2 illustrate the same results for AOIs PATTERN only. Group A (flat abstracted style) triggered a lower fixation density (A mean: 0.668 SD: 0.111) and higher average fixation duration (A mean: 302.757 SD: 70.335 ms) than the other two groups. Group B (tridimensional realistic style) present a low fixation density (B mean: 0.716 SD: 0.176) compared to group C (with B vs C t= 11.71 p< .001) and lower average fixation duration (B mean 271.534 SD: 58.66 ms) than the other two groups. The AOIs PATTERN in Group C (Klimt’s group) present, comparatively with the other two groups, a higher fixation density (C mean: 1.461 SD: 0.297) and low average fixation duration (C mean: 302.471 SD: 59.258 ms). As figures 6.1 and 6.2 best show, another type of comparison can also be made across Groups, this time considering the visual attention on AOIs FACE only. In this case Klimt’s Group seem to stimulate in the viewer the highest density (C Mean: 12.97 SD: 4.883) and average fixation duration (C Mean: 397.758 SD: 84.462 ms) on the sitter’s facial features.

**Figure 5.**
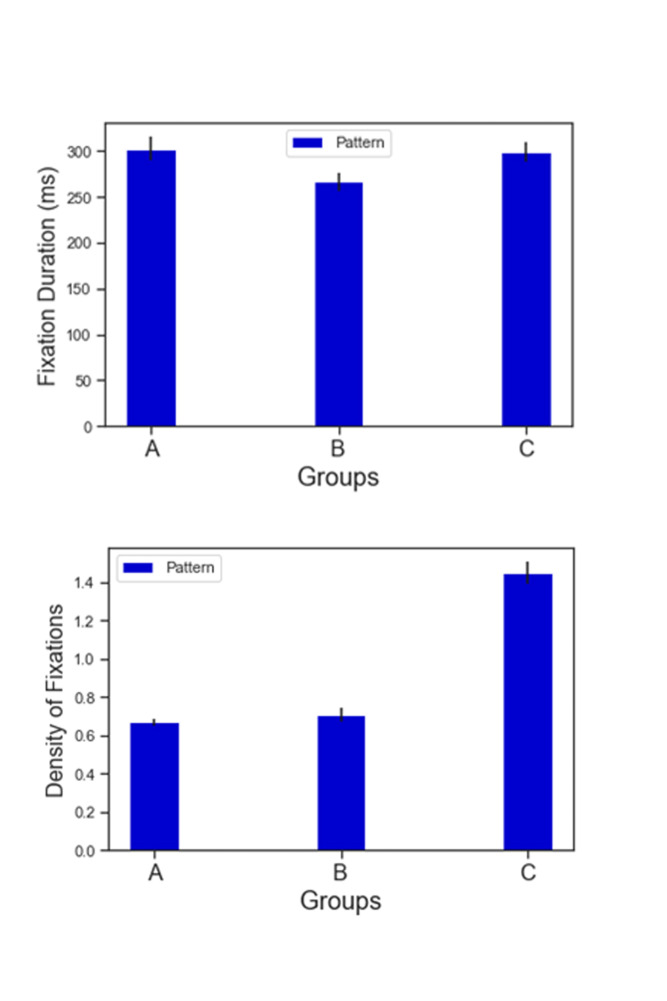
5.1, 5.2: Fixation duration (ms) and density of fixations across groups for AOI Pattern

**Figure 6.**
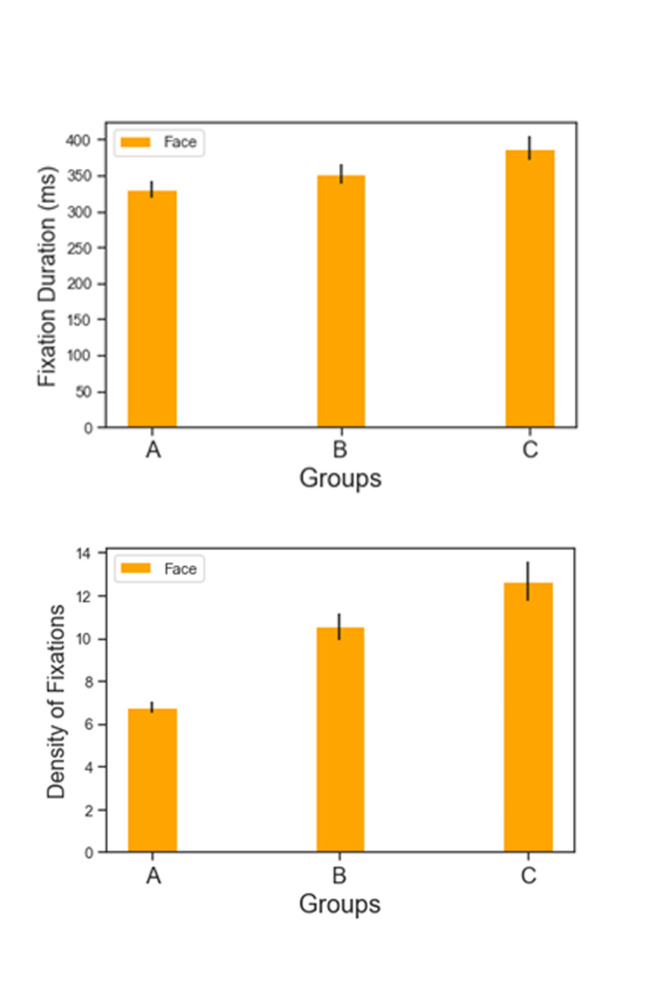
6.1, 6.2: Fixation duration (ms) and density of fixations across groups for AOI Face

## Discussion

Hypothesis 1 was confirmed: AOIs FACE presented consistently a higher density of fixation and higher average fixation duration than the AOIs PATTERN across all groups. This did not come as a surprise as it supports already cited research asserting that faces are always prioritised in scene perception. It does however prove that our selection of stimuli fit the theoretical criteria set before the experiment; all paintings allowed participants to focus on the sitter’s features and only secondarily on the ornament, regardless of the specific differences between one painting and another.

Hypothesis 2 was confirmed, with AOIs PATTERN of Group A and Group B being comparable for density of fixations and average fixation duration (As per Figure 5.1, 5.2). However, the relationship between the two differed from expectations. Average fixation duration for AOIs PATTERN was higher for Group A, modern paintings and lower for Group B, traditional. This is the opposite of what we claimed it would be. We imagined that realistic, pictorially tridimensional spaces would give viewers more contextual details to interpret; yet these results indicate that, given a similar density of fixation, participants spent less time looking at pictorial tridimensional ornament and more looking at abstracted ones – in fact, longer than the time they spent on Klimt’s. The “assembling gaze” we originally attributed to Klimt’s distinctive style seems to apply more to the abstracted ornament in modern portraits. A possible explanation to this could lie in our misattribution of *complexity*. While abstracted ornament is formally and semantically the simplest of the three in art historical terms – it is plain colour and brushstrokes, and shows little narrative function and no iconography – perhaps its very unfamiliarity is what requires us to explore it at length in order to understand what role it plays in the image.

This also implies that Hypothesis 3 was partly discredited. As shown in Figures 5.1 and 5.2 participants looked at both abstracted patterns (Group A) and tridimensional ones (Group B) less than Klimt’s (Group C), which was our first claim: Klimt’s ornament “absorbs” the gaze of the viewer more than the other styles of representation. Nonetheless the ornament of Group A stimulated the longest fixations: Abstracted ornament is the one triggering an “assembling gaze”. The last is the very outcome we had expected for Klimt, although in this case we imagined it would be caused by the evident contrast between realistic and flat elements. The difference between Group A and Klimt’s Group is actually relatively small, so we could, to an extent, still claim that this result applies to the ornament of both Groups: In other words, that the perception of Klimt’s bidimensional ornaments does not differ radically from the perception of bidimensional ornaments in modern paintings.

Nonetheless, a comparison with the AOIs FACE across Groups revealed one last dissimilarity between them. What emerges from this comparison is a more complex and perhaps more insightful understanding of what Klimt’s impact on the beholding experience could be; The results of group C in fact show two things:

1) That Klimt’s FACEs **and** PATTERNs stimulate the highest density of fixations across groups; This is considerable higher than Group A, marking a first difference with the perception of modern painting;
2) That the difference of average fixation duration between the two AOIs FACE and PATTERN is the highest across the three groups, 95ms (Group A = 28 ms; Group B: 82 ms). Group B seems also to report a difference between fixation duration for faces and patterns, but this can be explained by the general lack of interest in the AOI pattern altogether, given that it stimulates a very low fixation density: We give realistic ornament only a few rapid glances.

To sum up these results, we look at Klimt’s Faces the most and for the longest time; perhaps this is because the bidimensional ornament emphasizes, by contrast, the realism and characterization of facial features. Even more surprisingly, we look at Klimt’s ornament the most in the three Groups; however, our eyes are repeatedly performing fast looks on the canvas when we do so. This difference in fixation duration between FACE and PATTERN is not present in the two other Groups and could also be attributed to the juxtaposition of bidimensional and pictorially tridimensional elements coexisting in the same painting, requiring different cognitive efforts.

## Conclusions

This experiment was designed to test the validity of two different art historical claims; the first, that compared with other portraits, Klimt’s artworks elicit a unique response in the viewer. The second, that this response implies “absorbing” the gaze consistently (gathering a high share of fixations) and visually “assembling” his paintings piece by piece (triggering long fixations). The results show that compared with the other two groups, Klimt is leading our eyes more to faces and patterns, less to everything else in the picture. The ornament *and* the sitter gather our attention: We can therefore say that Klimt’s style does “absorb” our gaze more than other styles employed by his contemporaries to represent the same subject matter. As far as visually “assembling” his pictures, we had imagined that Klimt’s patterns, which are bidimensional and in strong contrast with the realistic facial features of the sitter, would have required long fixations to be identified and processed cognitively. This turned out not to be the case; the fixations are rather short, and the difference between fixation duration for Face and Pattern AOI is the highest of the three stylistic groups. This suggests that when looking at Klimt’s portraits our eyes perform alternately longer and shorter fixations. Rather than slowly “assembling” the painting, we could say that our eyes are moving in “scattered” looks.

Even if not as we imagined, it seems possible to suggest that we do have a specific physiological response to style considered characteristic or typical of Gustav Klimt, which do not apply to other modes of representation; moreover, we can also assert that this behavior supports what art historians have for so long described, discussing the coexistence of realistic and flattened elements as a dichotomy. We do not perceive tri- and bidimensional features equally; a correspondence exists between our eye-movements and the, so far only imagined, two-fold perception of “art” and “life” in Klimt’s pictures: This two-fold perception can be translated as the coexistence of long gazes, for faces, and scattered looks, for patterns.

## Limitations and Further research

The study was performed only with female participants between 18 and 30 years old. When expanding further on the topic, it would be important to understand whether we can obtain the same results with a more diverse group of participants, testing whether age, gender or sexual orientation are variables of meaningful impact in the aesthetic experience of Klimt’s art.

While only one person explicitly mentioned the contrast between rendering of facial features and rendering of ornament in the questionnaire, a large amount of our participants was conscious or became conscious of the characteristics building the basis of the study – namely, that Klimt’s rendering of ornament is the artist’s defining characteristic. This was not something we could or wanted to prevent. While it was important that participants were naïve to the aim of the experiment, our initial assumption was that Klimt’s style is widely recognizable. Testing whether our perception of Klimt differs from the perception of other paintings *because* we recognize his style goes beyond the scope of this paper. Researchers willing to expand on this aspect could conduct the same experiment with participants who are completely unaware of Klimt’s art, with different cultural backgrounds or exposed to a different artistic heritage.

On a final note, this investigation differs from most eye-tracking studies in that it employs art historical criteria to define and scrutinise categories of images. This implies a variety of limits: The main one is that Stimuli differ one from another in respect to several measurable characteristics (hight and width, angle and surface of AOIs). Nonetheless, however fluid the categorisation through style can sound compared with one based on objectively, quantifiable variables, experts and connoisseurs still identify artists by such categories – in art writing, style remains a valid tool to translate the image into word. Our aim here was not so much to prove that one element singled out from its picture (such as the contrast between bi-and tridimensional features) can change the perception of all paintings, nor we attempted to isolate such element. Rather, we intended to draw a parallel between art historical literature and the behaviour of the eye, in order to understand to what extent can this parallel be considered meaningful. Researching further whether specific eye movement patterns are elicited by Klimt’s portraits is a step towards a more complex understanding of the art historical language and its domain. Klimt’s distinctive, well-discussed and analysed art lends itself perfectly to the challenge of expanding this type of discussion.

## Ethics and Conflict of Interest

The authors declare that the contents of the article are in agreement with the ethics described in http://biblio.unibe.ch/portale/elibrary/BOP/jemr/ethics.html and that there is no conflict of interest regarding the publication of this paper.

## Acknowledgements

We wish to thank Flora Bakondi and Judith Herunter for their help, respectively, in the data gathering and revision process of this study.

## Appendix

### List of paintings employed in the experiment (Artist, Title, Material, Dimensions, Date, Location of original artworks)

**Table 1:** Group A

Edgar Degas	Portrait of a Young Woman	Oil on canvas	27.3 x 22.2 cm	1885	The Metropolitan Museum of Art	New York
Vincent Van Gogh	Adeline Ravoux	Oil on fabric	50.2 x 50.5 cm	1890	The Cleveland Museum of Art.	Cleveland
Henri de Toulouse-Lautrec	The Streetwalker	Oil on cardboard	64.8 x 53.3 cm	1891	The Metropolitan Museum of Art	New York
Pablo Picasso	Femme dans la Loge	Oil on canvas	81 x 60 cm	1901	Kunstmuseum	Basel
John Duncan Fergusson	Le Voile Persan	Oil on canvas	51.2 x 45.9 cm	1910	Hunterian Museum	Glasgow
Jalmari Ruokokoski	Love	Oil on canvas	58.5 x 55 cm	1910	Finnish National Gallery.	Helsinki
Gustav Klimt	Lady in White	Oil on canvas	70 x 70 cm	1918	Galerie Belvedere	Vienna

**Table 2:** Group B

Jean Auguste Dominique Ingres	Madame Jacques-Louis Leblanc	Oil on canvas	119.4 x 92.7 cm	1823	The Metropolitan Museum of Art	New York
Friedrich Amerling	The Young Eastern Woman	Oil on fabric	106.5 x 90.5 cm	1838	The Cleveland Museum of Art	Cleveland
William Morris	La Belle Iseult	Oil on canvas	71.8 x 50.2 cm	1858	Tate	London
William Holman Hunt	Fanny Waugh Hunt	Oil on canvas	104 x 73 cm	1868	Toledo Museum of Art	Toledo
Gustav Klimt	Portrait of Marie Breunig	Oil on canvas	155 × 75 cm	1894	Galerie Belvedere	Vienna
Felix Vallotton	Portrait de Gabrielle Vallotton	Oil on canvas	81 x 65 cm	1908	Clemens Sels Museum	Neuss
Édouard Vuillard	Lucy Hessel	Oil on board	88 x 67.9 cm	1924	Private Collection	

**Table 3:** Group C

Gustav Klimt	Portrait of Emilie Flöge	Oil on canvas	171 x 84 cm	1902	Wien Museum.	Vienna
Gustav Klimt	Margaret Stonborough-Wittgenstein	Oil on canvas	179.8 x 90.5 cm	1905	The Bayerische Staatsgemaeldesammlungen	Munich
Gustav Klimt	Portrait of Fritza Riedler	Mixed media on canvas	153 x 133 cm	1906	Galerie Belvedere	Vienna
Gustav Klimt	Portrait of Adele Bloch-Bauer	Mixed media on canvas	140 x 140 cm	1907	Neue Galerie	New York
Gustav Klimt	Hope II	Mixed media on canvas	110.5 x 110.5 cm	1908	MoMA	New York
Gustav Klimt	The Dancer	Oil on canvas	178 x 198 cm	1918	Leopold Museum	New York
Gustav Klimt	Johanna Staude	Oil on canvas	70 x 50 cm	1918	Galerie Belvedere	Vienna
